# Genome-driven cell engineering review: *in vivo* and *in silico* metabolic and genome engineering

**DOI:** 10.1042/EBC20180045

**Published:** 2019-06-26

**Authors:** Sophie Landon, Joshua Rees-Garbutt, Lucia Marucci, Claire Grierson

**Affiliations:** 1 BrisSynBio, University of Bristol, Bristol BS8 1TQ, U.K.; 2 Department of Engineering Mathematics, University of Bristol, Bristol BS8 1UB, U.K.; 3 School of Biological Sciences, University of Bristol, Life Sciences Building, Bristol BS8 1TQ, U.K.; 4 School of Cellular and Molecular Medicine, University of Bristol, Bristol BS8 1UB, U.K.

**Keywords:** Genome Engineering, in-silico, Metabolic Engineering, metabolic models, whole-cell models

## Abstract

Producing ‘designer cells’ with specific functions is potentially feasible in the near future. Recent developments, including whole-cell models, genome design algorithms and gene editing tools, have advanced the possibility of combining biological research and mathematical modelling to further understand and better design cellular processes. In this review, we will explore computational and experimental approaches used for metabolic and genome design. We will highlight the relevance of modelling in this process, and challenges associated with the generation of quantitative predictions about cell behaviour as a whole: although many cellular processes are well understood at the subsystem level, it has proved a hugely complex task to integrate separate components together to model and study an entire cell. We explore these developments, highlighting where computational design algorithms compensate for missing cellular information and underlining where computational models can complement and reduce lab experimentation. We will examine issues and illuminate the next steps for genome engineering.

## Introduction

Synthetic biology is the rational design and engineering of cells and cellular systems using genetic manipulations [1,2]. It is divided into three fields [3]: DNA-based device construction (production of functioning biological components to be inserted into cells), synthetic protocell development (construction of rudimentary representations of living cells), and genome-driven cell engineering. For more about DNA-based device construction principles see Brophy and Voigt [4], and for an introduction to protocell development see Dzieciol and Mann [5]. In this review, we will focus on genome-driven cell engineering (see Box 1 for key terms).

Box 1 **Key terms**

**Genome engineering:** Extensive and intentional genetic modification of a replicating system for a specific purpose [19].
**Minimal genomes:** Reduced genomes containing only the genetic material essential for survival, with an appropriately rich medium and no external stresses. No single gene can be removed without loss of viability [20].
**Recoded genomes:** Genomes with codon/s that have been freed, substituting codons for synonymous codons that encode the same amino acid, so that they can be assigned to new functions [21,22].
**Platform cell/cell factory/chassis (interchangeable):** A bacterial species that can efficiently convert raw materials into a product of interest, through genome engineering or hosting genetic components [6,23–26].
**Multiplex gene editing:** Simultaneous introduction of multiple distinct modifications to a genome [27].
**Algorithm:** Series of steps or rules to attempt to solve a problem, often implemented in a computer.
**Model:** Mathematical description of a system.
**Metabolic flux:** Metabolic reaction rate (i.e. turnover of molecules through a metabolic reaction).
**Flux vector:** A vector where each element corresponds to the metabolic flux of a reaction in the model.
**Genome-scale biological models:** Category of models containing: metabolic models, transcription regulatory networks, protein–protein interaction networks, integrated cellular models, and whole-cell models [11].
**Genome-scale metabolic models:** Models representing all active reactions in a cell/organism as a matrix of stoichiometric coefficients of each reaction, and linking reactions with gene products that catalyse them. Abbreviated to GSMMs or GEMs [28,29].
**Whole-cell models:** Describe the life cycle of a single cell, modelling individual molecules and interactions, and includes the function of every known gene product [13].

Genome-driven cell engineering encompasses both metabolic engineering (control of cellular production processes) and genome engineering (production of minimal genomes, recoded genomes, and cellular chassis/factories). It encompasses diverse types and scales of genetic modifications and underscores the genome as the major driver of cellular events [3].

Metabolic engineering attempts to improve titre, accumulation rate, and yield of a specific metabolite, often from microorganisms in an industrial setting [6]. Genome engineering attempts to: understand (comprehending biological systems by trying to engineer them [7], e.g. minimal genomes), reduce risks (restricting bacteria to specific media [8], e.g. recoded genomes), and improve metabolite production (e.g. ‘optimal’ chassis cell development for metabolite production [9]).

Here, we review metabolic engineering and genome engineering from both biological and computational perspectives. Metabolic engineering, with established *in silico* design and simulation (hundreds of models, tens of algorithms) [10,11] and *in vivo* construction methodologies (hundreds of strains of several bacterial species) [12], could inform the future of genome engineering, given the development of whole-cell mathematical models [13], genome design algorithms [14,15], and CRISPR-cas9 gene editing techniques [16–18]. Finally, we examine issues and the next steps for genome engineering.

## Metabolic engineering *in vivo*

Metabolic engineering enhances the production of native or introduced metabolites, often in a microbial strain [6]. Genetic edits are used to introduce or modify the required pathway and take control of core metabolism, cellular regulation, and stress responses [6,12]. Applications are wide ranging, including fuels, feed additives, and pharmaceuticals [12,30], and determine the most appropriate microorganism for production (see Table 1).

**Table 1:** A selection of microorganisms used for metabolite production

Microorganism	Primary feature	Applications	Product examples	Strain examples
*Escherichia coli*	Variety of tools/knowledge	Exploratory production, established industrial strain	1-3-Propanediol, 1-4-Butanediol, butanol, insulin, limonene, l-threonine, l-serine, PHAs, propane, succinate	Based on K-12 and B ancestor strains. Derivatives of MG1655, W3110, BW25113. Specific strains: BL21 Rosetta, DH1, ATCC 31884, DH10B
*Bacillus subtilis*	Efficient secretion systems	Protein production	Amylases, bacitracin, biotin, cellulosome, chiral stereoisomers, cobalamin, glucanases, guanosine, laccases monophosphate, riboflavin, subtilisin, vitamin B_6_	Protease-defective mutants: WB600, WB800. Specific strains: 168, RH33, BSUL08, 1A1, E8, KU303
*Pseudomonas putida*	Chemical resistance	Harsh conditions and toxic product production	3-methyl-catechol, anthranilate, cinnamic acid, PHAs, phenol, *o*-cresol, styrene, terpenoids, vanillate	Specific strains: KT2440, EM42, Gpo1, S12
*Cyanobacteria*	Photosynthetic	Light-driven production	1-butanol, 1,3-propanediol, bisabolene, ethanol, farnesene, isoprene, isopropanol, PHAs	Specific strains: PCC- 6803, PCC-7942. PCC-7002

Information collated from Nielsen and Keasling [12] Calero and Nikel [6], Gu et al. [34], and Pontrelli et al. [33].

Only a small number of microorganisms are ‘industry ready’, such as: *Escherichia coli (E. coli), Bacillus subtilis (B. subtilis), Streptomyces sp., Pseudomonas putida, Corynebacterium glutamicum* [6], *Saccharomyces cerevisiae* and *Aspergillus niger* [12]. Requirements for industrial microorganisms are simple nutritional needs, fast and efficient growth, high resistance to extreme physical and chemical conditions, and efficient secretion systems [6]. Also required are sufficient genetic and metabolic knowledge and a range of genetic tools (e.g. promoters and terminators with varying expression levels, and well-characterised plasmids for precise manipulations). Due to the development of CRISPR-cas9 gene editing tools [31,32], a number of novel bacterial species are now usable, including *Vibrio natriegens* (has the shortest known doubling time, at 15 min), and *Roseobacter* and *Halomonas* (marine species with salt tolerance) [6]. Metabolic engineering has recently been reviewed for *E. coli* [33] and *B. subtilis* [34].

The metabolic production pathway is constructed, reconstructed, or tweaked in the strain, and can then be iterated upon to produce improvements in titre, rate, and yield. There are six strategies [34] for improving these: (i) modular pathway engineering, which divides up the production pathway to produce and combine modules with different expression levels [35]; (ii) cofactor engineering, in which metabolic flux to the desired products is enhanced through gene edits that alter non-protein cofactor levels [36]; (iii) scaffold-guided protein engineering, where the spatial locations of proteins in the cell are modified to increase local concentrations of intermediates [37]; (iv) transporter engineering, which improves the import of substrates [38] and export of products [39]; (v) dynamic pathway analysis, which identifies unknown network interactions and promotes or suppresses them to increase levels of product [40]; and (vi) evolutionary engineering, which mimics natural evolutionary approaches to produce greater amounts of product [41–43].

The development of an ‘industry ready’ strain takes several years and is costly. Strains for Artemisinin and 1,3-propanediol production took 10 years and $50 million, and 15 years and $130 million, to develop respectively [12], though sales of metabolic products are expected to reach $6.2 billion by 2020 [44]. The time and cost is due to complex interactions and regulation in metabolism. Metabolite intermediates and products can cause toxicity and act as inhibitors of other reactions, or be misrouted or modified by unrelated enzyme reactions, leading to decreasing titre, rate, and yield [6].

Recently, the availability of accurate, genome scale metabolic models, refined with data captured using omics technologies, has begun to overcome these limitations and support rounds of *in silico* design and *in vivo* construction. [6,34].

## Metabolic engineering *in silico*

### Constraint-based metabolic models

Recent advances have allowed the reconstruction of genome-scale metabolic networks, and subsequently *in silico* models which can predict cellular phenotypes. Metabolic models are based on biological knowledge and experimental data; metabolic kinetic models describe how metabolites vary in time using differential equations, while metabolic constraint-based models formalise behaviour at steady-state (i.e. metabolite production is equal to metabolite loss) [45]. There are currently very few genome-scale kinetic models, due to the lack of experimental data for enzyme rate parameters, but the static nature of constraint-based models means they require significantly fewer parameters to construct, and so are more widely used. For this reason, we will mainly review constraint-based models.

**Figure 1:**
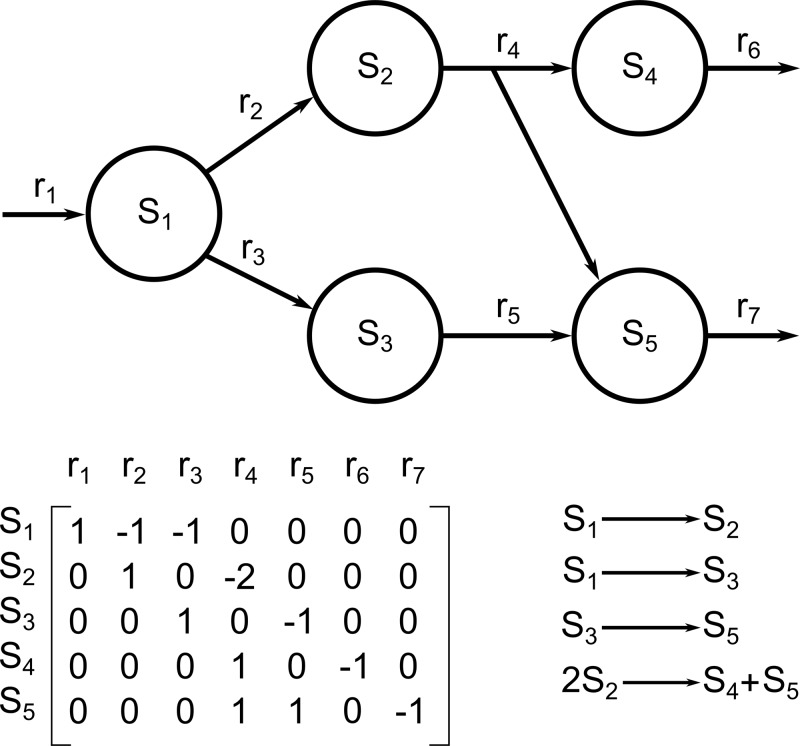
A toy metabolic network S_i_ are the substrates and r_i_ are the reaction rates. The network can be represented as a stoichiometric matrix (whose columns and rows correspond to reactions and metabolites, respectively), and a system of equations.

Genome-scale metabolic models (GSMMs/GEMs) aim to form a solution space of flux values for each reaction in a metabolic model (see Orth et al. [46] for more details), or can give insight into the behaviour of the system through network analysis. These models consist of a stoichiometric matrix and a set of biologically feasible constraints for reactions (Figure 1).

**Figure 2:**
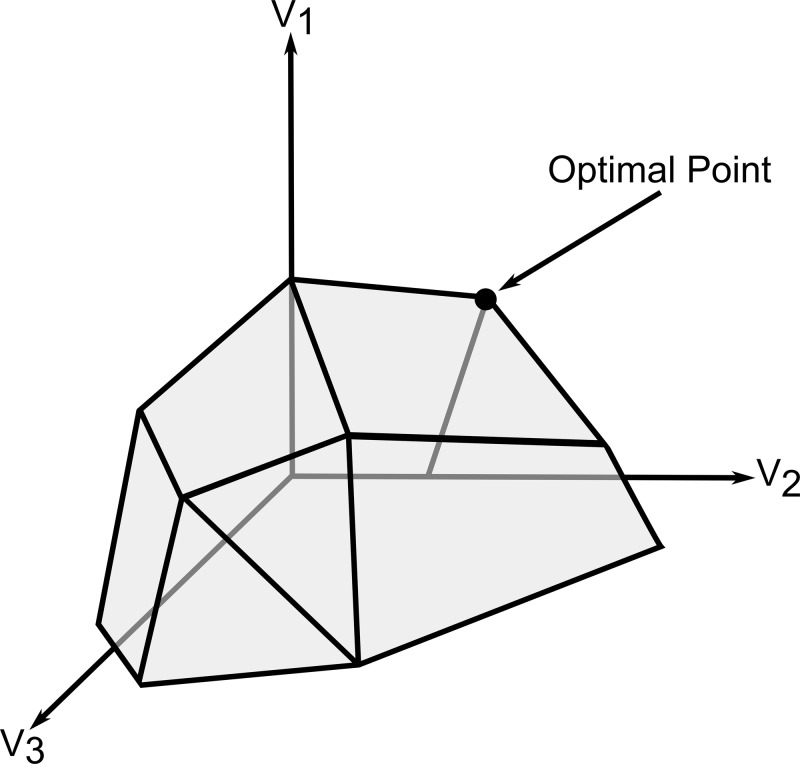
Creation and timeline of bacterial GEMs over the past two decades More complex genome-scale computational models (such as metabolic and macromolecular expression (ME) models and the first whole-cell model), modelling automation tools (ModelSEED and CarveMe) and the ME software frameworks COBRAMe are also included. 2001 did not see any models created.

There are currently 113 bacteria, 57 eukaryote and 8 archaea curated GEMs available at UCSD Systems Biology [47], see Figure 2. Tools for automatically generating GEMs have been developed, first modelSEED [48] and the most recently CarveMe [49]. CarveMe begins with a universal model consisting of 2383 metabolites and 4383 reactions, formulated from the BiGG database [50,51]. This can be stripped down to become a metabolic model for any specific organism, using its annotated genome. There are multiple other automation tools available: AuReMe [52], Merlin [53], MetaDraft [54], Pathway Tools [55], and Raven [56], which have been recently reviewed [57].

### Exploring and analysing the steady-state solution space

**Figure 3:**
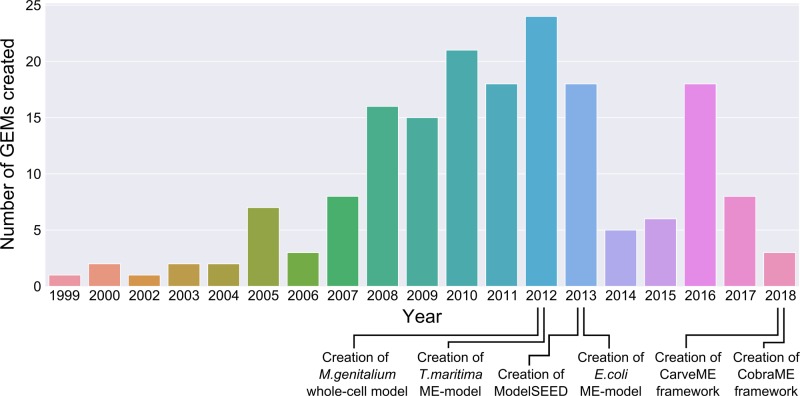
A schematic of the feasible region found through constraint-based modelling Where v_i_ are fluxes of the system and form a flux polyhedron. The flux values that optimise the objective function can be found by looking at the extreme edges of the polyhedron, and selecting the point that fits the optimisation criteria.

There are numerous ways to simulate and analyse metabolic models, depending on the desired information. Elementary flux modes (EFMs) can be found, based on the stoichiometric matrix—these are the set of non-decomposable reactions that trace input metabolites to output metabolites which can be used to break a metabolic network into its component pathways [58]. In the context of metabolic engineering, these can be analysed to choose reactions to disrupt in order to direct cell resources towards specific metabolites. Alternatively, the fluxes through each reaction when the system is at steady state can be found, either through Monte Carlo sampling [59] or, more commonly, by flux balance analysis (FBA). The solution space of the system at steady-state can be found by combining the constraints on the system to form a region which can be analysed, as shown in Figure 3.

To perform FBA, first an objective function is defined that can maximise or minimise the flux through any reaction in the system. When simulating a wild-type unicellular organism in the exponential growth phase, the rate of biomass production is maximised as a proxy for cellular fitness [60], and used as the objective function. This is formulated as a ‘pseudoreaction’, meant as the sum of the different biomass components (e.g. amino acids, fatty acids, vitamins, and cofactors); the flux through which is maximised. Other objective functions can be used for different purposes: for example, minimisation of ATP production [61], or optimisation of several reactions in parallel.

FBA optimisation generates flux vectors that are used to optimise a defined objective function. The flux vectors give insight into the dynamics of the system when equilibrium is reached, indicate which pathways the metabolites are involved in, and can also predict the behaviour of the simulated cell when grown in different culture conditions.

FBA is available in the COBRA (constraint-based reconstruction and analysis) toolbox for Python or MATLAB numerical computing environments. For an overview of the COBRA ecosystem see Lewis et al. [62].

### Alternative methods to optimise the solution space

GEMs can be used for metabolite optimisation by analysing the effects of adding or removing genes. FBA can be used to directly calculate fluxes in cells with gene knockouts, or FBA wild-type fluxes can be used as input for other methods to calculate fluxes after gene knockouts: MOMA (minimisation of metabolic adjustment [63]) and ROOM (regulatory on/off minimisation of metabolic flux [64]). While FBA picks a solution that optimises a given objective function, MOMA and ROOM output a solution which minimises the distance between the wild-type and the altered metabolism fluxes, or the number of changes in flux respectively. Given that a strain after a knockout is not a result of evolution, the assumption of the FBA objective function mimicking evolution may no longer be relevant and so both MOMA and ROOM account for the cell behaviour immediately after *in vivo* knockouts, which can be different from cell behaviour over a longer time scale [65].

### Predicting gene essentiality using GEMs and algorithms

Metabolic models can be used also to predict gene essentiality—gene knockouts can be simulated and then cell survival assessed based on the end production of biomass (i.e., if the simulation results in zero biomass production, the cell is presumed to be dead and therefore the knocked-out gene is essential). This has successfully been shown for *E. coli* strains such as MG1655, where gene deletions simulated *in silico* correctly predicted the essentiality of 86% of single gene deletions [66]. Similar gene essentiality testing has been performed with FBA models of other organisms, including *Helicobacter pylori* [67], *Saccharomyces cerevisiae* [68], *B. subtilis* [69], and *C. glutamicum* [70], showing the accuracy of these models.

### Further Development of GEMs

GEMs can also act as a springboard for more detailed cellular models that take into account transcription processes. These extended models, still in early stages of development, have not yet improved the accuracy of standard FBA models [49]. More recently, macromolecular expression (ME) has been incorporated (ME-models) to integrate tRNA charging, transcription, and translation reactions with metabolic reactions. The metabolic reactions are coupled with the macromolecular synthesis reactions of the enzymes that catalyse them, and the synthesis reactions for transcription and translation components (e.g. mRNA and proteins) are formed from the metabolic biomass production. The COBRAMe framework [71] aids generation of ME models, for example an *E. coli* model (iJL1678b-ME) that is more efficient than the first *E. coli* ME model (iOL1650-ME [72]), containing 1/6 variables and solving in 1/36 of the time.

The main limitation to this approach is the lack of well-curated databases: while genome and gene product information can be retrieved (e.g. using KEGG [73] and Genbank [74]), no single database contains rate parameters for transcription and translation, necessary for ME model parameterisation [75].

### Metabolic engineering applications: constraint-based modelling and metabolic network analysis

As discussed above, GEMs can be used to study wild-type cell behaviour, as well as investigate the effects of gene knockouts. Another application is for metabolic engineering, where algorithms can use GEMs to predict genes within the model to knockout, amplify or inhibit, in order to produce a pre-defined goal of overproduction of some metabolite.

### Metabolic engineering using Elementary Flux Modes (EFMs)

As well as providing scope for analysis of metabolic networks, EFMs can be used to isolate pathways that can be disrupted to force a cell to overproduce a metabolite. As EFMs find minimal pathways through the metabolic network, the paths from an input substrate to a chosen metabolite and its efficiency (i.e. the stoichiometry and length of the chain of reactions) can be found. Competing reaction pathways can then be found and removed, thereby producing a streamlined strain with minimal functionality. Although this process involves significant computational power, especially for genome-scale models, it has been shown to have success in improving lysine production in *C. glutamicum* [76].

### Metabolic engineering using nested linear programming-based methods

Whereas FBA, MOMA and ROOM take gene modifications as their input and output a corresponding flux distribution, other algorithms designed specifically for metabolic engineering take a metabolite (other than biomass) as their input, and output a set of gene modifications that optimise its production.

For example, OptKnock maximises the production of a specified metabolite and biomass by deleting genes to re-route metabolites through certain reaction pathways [77]. Genome designs for the overproduction of succinate and lactate *in silico* using OptKnock were consistent with laboratory results [77].

**Figure 4:**
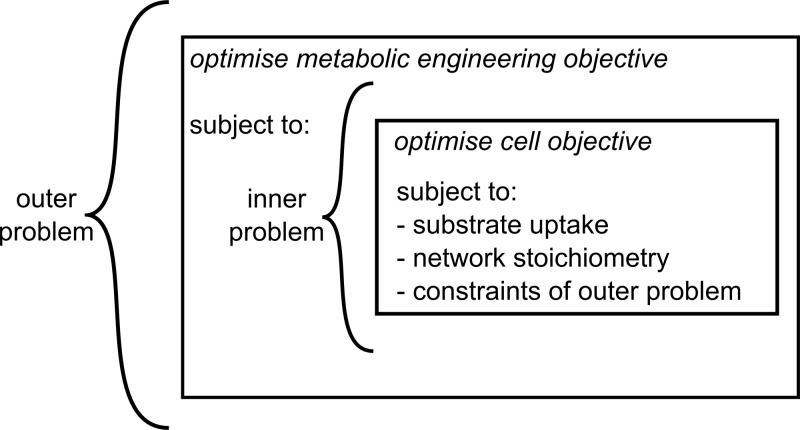
Bilevel linear programming The nested structure of the bilevel linear programming algorithms, where the inner problem optimises for a cellular objective function and the outer problem optimises for some metabolic engineering objective.

Several algorithms for metabolite optimisation use linear programming and couple cell growth and biochemical production using bilevel mixed-integer linear program (MILP), a nested framework where an outer optimisation problem (e.g. maximise metabolite) is constrained by another inner optimisation problem (e.g. maximise biomass), as shown in Figure 4. This two-stage optimisation problem can be intractable; therefore, OptKnock [77], OptORF [78], and RobustKnock [79] reduce the bilevel problem to a single level problem using duality theory (i.e. an approach that enables optimal solutions of two problems to be found by setting their objectives equal to one another).

Alternatively, ReacKnock [80] uses Lagrangian multipliers (a process for non-linear optimisation) to allow specifically equality constraints, which can then reformulate the bilevel problem into a single level problem [81].

EMILiO [82] also uses linear programming, but iteratively: it begins by pruning the metabolic network to select a subset of the flux constraints that will maximise the metabolite production rate, then it prunes the subsets to minimise the number of reaction modifications. It will then output knockout, activation or inhibition modifications to produce the desired metabolite overproduction.

### Optimising metabolism using reaction flux regulation

OptReg [83] and OptForce [84] output reactions to be up-regulated or down-regulated to create a desired flux distribution. They first calculate upper and lower bounds for every reaction flux in the system by iteratively changing the objective function to maximise and minimise each reaction, then compare these ranges to the flux distribution of a metabolism that overproduces a targeted metabolite. It is possible to identify the reactions that require regulation to transform their behaviour into that of the system that overproduces the targeted metabolite. OptForce has the addition of predicting knockouts as well as regulation changes, and also minimises the amount of interventions needed to achieve metabolite overproduction.

### Optimising metabolism using metaheuristic algorithms

Other approaches are based on metaheuristic algorithms [85] (Table 2), which are high-level methods used to search a solution space. They are particularly powerful when sampling a large solution space using incomplete information, and often use optimisation methods that contain a degree of stochasticity. Multiple *E. coli* strain GEMs contain over 2000 reactions, but the possible combinations for only five gene knockouts is 10^15^, making the solution space huge. However, metaheuristic algorithms do not guarantee a globally optimal solution and they require significant computational power.

**Table 2:** Metaheuristic algorithms for analysis of metabolic models and metabolic engineering

OptGene (Patil et al., 2005) [86]	Uses a genetic algorithm (the outer problem) to iteratively run FBA (the inner problem) with different knockout combinations to maximise metabolite production
RegKnock (Xu, 2018) [87]	Uses a genetic algorithm and a regulatory FBA model [88] for the inner problem, where extra constraints are placed on the system to model gene regulation events to maximise chosen metabolite production
FOCuS (Mutturi, 2017) [89]	Divides the total reactions into smaller groups, which are individually evaluated, as a pre-processing step, followed by a combination of flower-pollination algorithm [90] and clonal selection algorithm [91] to maximise metabolite production
GACOFBA (Salleh et al., 2015) [92]	Uses a combination of ant colony optimisation and a genetic algorithm as the outer problem to maximise metabolite production

### Optimising metabolism using non-native reactions and neighbourhood searching

Additional approaches for algorithmic metabolite optimisation include OptStrain [93], which searches through the KEGG database to find non-native reactions to add to a GEM to optimise metabolite production.

Genetic Design through Local Search (GDLS) [94] iteratively searches through possible solutions (e.g. knockout sets) that differ from the starting conditions using a neighbourhood search (a metaheuristic method for searching over the solution space by exploring solutions in the ‘neighbourhood’ of the current solution), and stores the best solutions in each iteration. Whereas bilevel linear programming approaches scale exponentially, the runtime for GDLS scales linearly with the number of knockouts, making it more efficient.

### Metabolic models and algorithms summary

**Figure 5:**
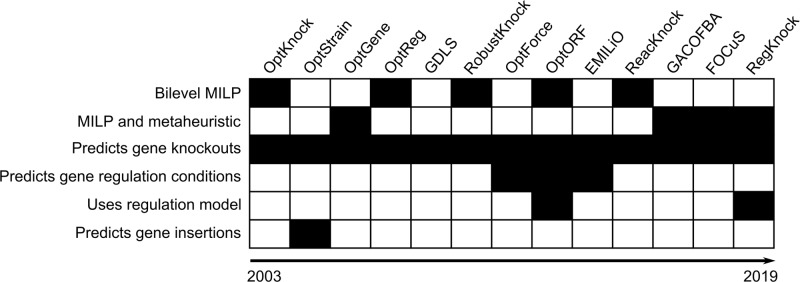
Comparison of metabolic engineering algorithms/frameworks features Black rectangles indicate feature presence, white rectangles indicate absence.

Choosing an algorithm to use (Figure 5) has to take into account the experimental methodologies available (i.e. the number of knockouts which can be performed), and the available computational power (significantly higher for metaheuristic algorithms). Also, the validity of results has to be critically considered given the accuracy of reaction databases used by some algorithms (e.g. OptStrain), and possible unrealistic results in simulated flux distributions when using entirely stoichiometric representations of metabolic pathways.

## Genome engineering *in vivo*

Genome engineering is the production of modified genomes using either a prescriptive genome design or a clear laboratory-based algorithm to design gene edits, and accurate genetic tools that can be used repeatedly.

**Figure 6:**
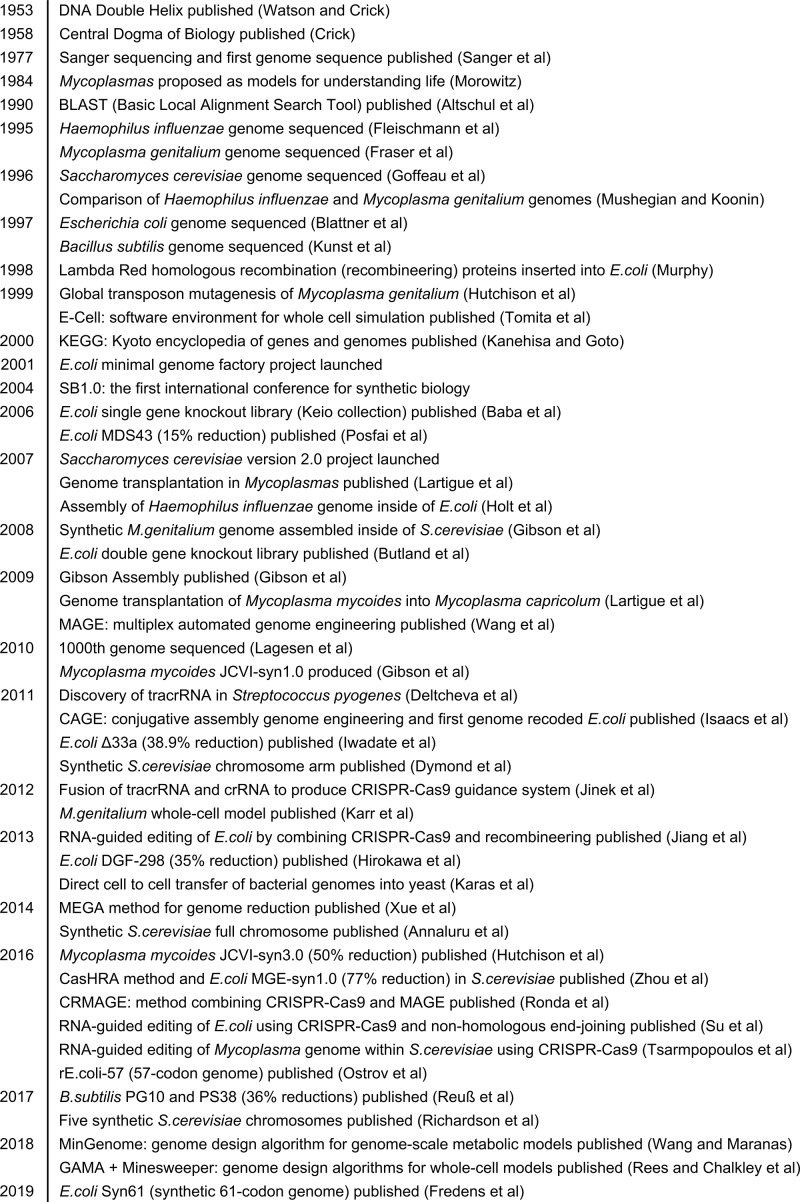
An incomplete history of genome engineering in microorganisms

Genome engineering builds on historical gene essentiality research (see Figure 6). The sequencing of small bacterial genomes [95,96] led to comparative genomics, initially between pairs of bacteria [97], then including greater numbers of bacteria as genome sequencing increased, which led to the development of minimal gene sets [97–99]. However, as the number of microorganisms increased, the number of shared genes decreased: by the thousandth genome sequenced only four genes were shared across all sequenced bacteria [100]. This trend is true even among closely related species, 20 *Mycoplasma* strains were found to share only 196 genes [101]. This is due to non-orthologous gene displacements (NOGDs), independently evolved or diverged proteins that perform the same function but are not recognisably related [20,97]. This comparative work continues to be built on computationally, analysing the growing number of genomic datasets for key features that could match NOGDs (see persistent gene concept [102]); nevertheless, genome engineering has moved to a species-specific focus.

Single gene knockout studies (implemented by systematic removal, inactivation, transposon mutagenesis, and antisense RNA [103]) are still used to provide an initial assessment of gene essentiality, but further work is required, as gene essentiality has been shown to depend both on the environmental context (i.e. how cells are grown) [104] and genomic context (i.e. what other genes present) [105].

Consequently, non-essential and essential classifications have been expanded to no essentiality, low essentiality, high essentiality, and complete essentiality [105], with other important classifications for genome engineering including quasi-essential (removal reduces growth rate substantially [106]), synthetic lethal (removal can kill the cell depending on the presence/absence of related genes [107,108]), and synthetic rescue (multiple genes that are essential individually, that can be removed together [109,110]). This redefinition of essentiality has underlined the existence of multiple minimal genomes for individual bacterial species, depending on environmental conditions [26,105], and the selection of redundant genetic pathways in the cell [14].

Research for understanding (minimal genomes) and production (chassis development) (see Table 3) both involve large numbers of gene/base pair deletions and use similar genetic tools. However, they differ in intent: no single gene can be removed without loss of viability in minimal genomes [20], whereas the cellular growth rate is maintained or promoted in chassis development. Additionally, minimal genome research focuses on protein-coding genes ignoring: essential promoter regions, tRNAs, small non-coding RNAs [26], regulatory non-coding sequences [103], and the physical layout of the genome [103,111], which are of interest to chassis development. Finally, bacterial species that do not have a use industrially are of use in minimal genome research. *Mycoplasma genitalium* only synthesises DNA, RNA, and proteins from imported precursors, in order to replicate itself [20], which it does slowly in a stress-free laboratory environment [106]; useful for understanding but not for industry.

**Table 3:** Genome-driven cell engineering examples

Genome Reductions		
Microbe	Reduction	Benefits
JCVI-Syn3.0 (Hutchison et al., 2016) [106]	50%	Smallest genome of any autonomously replicating cell. Has a doubling time of ∼180 min, four to five times faster than *M. genitalium* (12–15 h [20])
*E. coli* Δ33a (Iwadate et al., 2011) [113]	39%	-
*E. coli* DGF-298 (Hirokawa et al., 2013) [9]	35%	Better growth fitness and cell yield, in a rich medium, than the wild-type strain, and has a more stable genome
*B. subtilis* PG10 and PS38 (Reuß et al., 2017) [112]	36%	Subsequently used for production purposes, as has traits that are favourable for producing ‘difficult-to-produce proteins’, overcoming several bottlenecks (secretion process and unstable product) [122]
*E. coli* Δ16 (Hashimoto et al., 2005) [123]	30%	-
*B. subtilis* MGIM (Ara et al., 2007) [124]	24%	Little reduction in growth rate and comparable enzyme productivity
*E. coli* MGF-01 (Mizoguchi et al., 2008) [114]	22%	Better growth rate resulting in 1.5-fold cell density and 2.4-fold greater threonine production compared with the wild-type strain
*B. subtilis* MBG874 (Morimoto et al., 2008) [125]	20%	Extracellular cellulase and protease production were 1.7- and 2.5-fold higher. Production period was elongated and carbon utilisation improved
*E. coli* MS56 (Park et al., 2014) [126]	23%	Insertion sequence free, making it more genomically stable, predicted to increase production of recombinant proteins
*E. coli* MDS43 (Posfai et al., 2006) [127]	15%	Showed genome stabilisation and increased electroporation efficiency, comparable with *E. coli* DH10B. Subsequently used for production purposes: 83% increase in l-threonine production, compared with *E. coli* MG1655 with the same metabolic engineering [116]
**Genome Recoding**		
**Microbe**	**Modifications**	
32 *E. coli* strains (Isaacs et al., 2011) [8]	Replaced 314 TAG (stop) codons with TAA	
*E. coli* MG1655 (Lajoie et al., 2013) [22]	Replaced 321 UAG (stop) codons with UAA	
r*E. coli*-57 (Ostrov et al., 2016) [119]	Replaced 62214 instances of seven codons (UAG (stop), AGG and AGA (Arg), AGC and AGU (Ser), UUG and UUA (Leu))	
*E. coli* C123 (Napolitano et al., 2016) [128]	Replaced 123 rare AGA and AGG (Arg) codons from essential genes with 110 CGU conversions and 13 optimised codon substitutions	
*E. coli* MDS42 (Wang et al., 2016) [129]	Tested 1468 codon changes using REXER technology and GENESIS method	
*S. cerevisiae* Sc2.0 (Richardson et al., 2017) [130]	Replaced TAG (stop) codons with TAA	
*E. coli* Syn61 (Fredens et al., 2019) [131]	Replaced 18214 codons, TCG with AGC, TCA with AGT, TAG with TAA, using REXER technology and GENESIS method	

Of the largest scale reductions to date (see Table 3): *JCVI-Syn3.0* [106], and *B. subtilis* PG10 and PS38 [112] were produced for the purposes of understanding, and *E. coli* Δ 33a [113] and *E. coli* DGF-298 [9] were produced as chassis cells for production. Regardless of original intent, minimal genome reduction strains can have emergent beneficial properties [114,115] (see Table 3) in addition to the lower metabolic burden and increased metabolic efficiency produced by reducing gene numbers [116]. Additionally, the reduced internal biochemistry may interfere less with introduced external pathways [117], making for improved chassis cells. Two minimal genome reduction strains have been subsequently used for production purposes (see Table 3).

Research for reducing risks (genome recoding) substitutes synonymous codons (encoding the same amino acid) across an entire genome resulting in: virus resistance (viral replication relies on all 64 codons [21]), prevention of gene transfer [118], and increased translation efficiency [8]. It also produces a blank codon that can be repurposed for a novel function not commonly found in nature [8,21,119]. This incorporation of non-standard amino acid (NSAA) is a form of biocontainment, further reducing risk, as the organism is engineered to be dependent upon the presence of the synthetic NSAA to survive.

Genome recoding is possible due to the development of MAGE (multiplex automated genome engineering) [120] and CAGE (conjugative assembly genome engineering) [8,121], and subsequently REXER [129]. MAGE cyclically targets many genetic locations to conduct mismatches, insertions, deletions in a single cell or across a population of cells, maintaining high efficiency of up to ten targets at a time [120]. This leads to rapid and continuous generation of genetic diversity for strain and pathway engineering. CAGE is a complementary method, assembling modified genomic modules from individual cells into a single genome through cell to cell transfer, and has been used in combination with MAGE to systematically recode codons [8,121].

Combining genome engineering research together can give insights into what an ‘optimal’ cellular chassis could look like (see Table 4) and suggest research pathways going forward.

**Table 4:** Features of an optimal chassis for a wide range of applications

Feature	Description
Genetically stable	Removal of mobile DNA elements (e.g. insertion elements, transposases, phages, integrases, site-specific recombinases) [132]
Genomically recoded	Substitute codons to create blank codons for inclusion of new, non-natural amino acids [8], decreased likelihood of viral infection [21], and horizontal gene transfer [118]
Genome minimised	Removal of competing and unwanted metabolic pathways that divert the resources of the cell away from desired end products [19], resulting in increased capacity for and reduced impact of cellular burden [133,134], and greater robustness and energy efficiency [135]. Also reducing transcriptional regulatory interactions resulting in lower resistance to engineering efforts [132]. Additionally, allows exploitation of larger and optimal precursor pools [136]
Production efficiency	Simple nutritional needs, fast and efficient growth, and efficient secretion systems [6]
Robustness	Tolerance for extreme conditions [6] i.e. strength of cell membrane or wall and appropriate coping mechanisms [26]
Well understood	Sufficient knowledge of the organism’s genome and metabolism to produce accurate mathematical models and modularisation of metabolic pathways [26].
Developed tools	A range of established genetic tools for manipulation, including promoters and terminators with varying expression levels, and well-characterised plasmids, to enable titre, rate, and yield improvements and rapid and efficient tuning of genetic components [19]

## Genome engineering *in silico*

### Whole-cell models

The first whole-cell model [137] has been produced recently and is an important development for *in silico* cellular research, as the first integration of mathematical models to simulate an entire cell’s components. The recency is due to the immense complexity of individual cells. There are many well-characterised cellular subsystem models, such as ordinary differential equation (ODE) or network models for protein interactions [138], but combining different subsystems together has only been feasible in the last decade.

The *Mycoplasma genitalium* whole-cell model [13] consists of 28 linked submodels that simulate different cellular processes e.g. metabolism using FBA and cell division using ODEs. The model is implemented in MATLAB and produces large amounts of output data. Genes can be knocked out of the model, and environmental variables altered, so the cellular behaviour can be examined in various different situations.

A recent application of the *M. genitalium* whole-cell model is *in silico* genome reduction [14]. This is due to the ease and low cost of simulations (with the appropriate computational infrastructure) compared with *in vivo* experiments. Although modelling is never 100% accurate, it can help to shed light on unexplained phenomena and guide the design of lab experiments, producing research more efficiently [139]. However, even with a genome as small as *M. genitalium* (525 genes), the number of possible gene knockout combinations at genome-scale is of the order of ten [127], making simulating every knockout set unattainable due to time and computational constraints.

Algorithms to reduce the solution space can be used. Minesweeper and GAMA (Guess Add Mate Algorithm) [14] identified up to 165 genes that can be removed from the *M. genitalium* genome while still producing a dividing *in silico* cell. Minesweeper approximates a divide-and-conquer algorithm by knocking out gene sets of varying sizes, then combining sets that produced a dividing cell, generating knockout sets of greater size (thus a smaller genome). GAMA begins similarly, knocking out gene sets of varying sizes, followed by a genetic algorithm to combine these sets iteratively over multiple generations, to make the genome smaller.

These approaches are similar to the metaheuristic algorithms used for metabolic engineering, they are purely input/output dependent. These algorithms could potentially be applied to any model, regardless of its formulation, so theoretically scalable from the GEM level to the whole-cell model level. OptGene and GAMA both use a genetic algorithm to achieve their results, except with a different objective function. It is plausible that GAMA could be modified to maximise a metabolite at the whole-cell level, and equally possible that FOCuS and GACOFBA could be applied to whole-cell models for similar purposes or for genome minimisation.

Whole-cell models are a vital new approach for genome design. Combined with flexible algorithms (e.g. genetic algorithms [140] and ant colony optimisation [141]) they can suggest genetic modifications to produce organisms designed for specific purposes, while producing greater understanding of cellular processes and genetic interactions.

## Issues

There is a clear need for greater species-specific understanding of the metabolism and the genome. Even well-studied organisms (*B. subtilis* and *E. coli*) have genes with unknown functions and essentiality; bacterial genomes have on an average 33% genes of unknown function [142]. Of the genes with known functions, in most cases we only understand essentiality at the single or double knockout level [143,144]. Current genome reductions have had to identify synthetic lethal interactions as part of their reduction efforts, rather than being able to design around them. If we had a greater grasp of gene product interactions, enabling them to be accurately modelled, this could be avoided. We would also be taking steps towards a proposed end goal of genome design, combining modular components of different bacteria in a novel cell [24,145].

Another approach for genome-driven cell engineering, constructing bacterial genomes from scratch and inserting them into a host cell, is not currently possible in the majority of bacteria due to economic and technological constraints. Economically, bacterial genome production is too expensive for most institutes. Producing JCVI-Syn1.0 was estimated to cost ∼$40,000,000 [146]. Technologically, megabase-sized genomes can be constructed in yeast [147,148], however successful genome transplantation has only been demonstrated in a few *Mycoplasmas* [149–151].

Development of whole-cell models for genome engineering is time and cost intensive. The *M. genitalium* whole-cell model took 10-person years to build [152], resulting in the Karr Lab at Mount Sinai developing automation tools (Datanator and WC-Lang [153]) along the lines of automated tools for producing GSMMs.

Currently, genome engineering has not combined computational and biological research, due to how recently the required tools were developed [13–16] and the difficulty of working with *M. genitalium* in the lab [106]. This is set to change with the upcoming publication of an *E. coli* whole-cell model, as well as other whole-cell models [154]. In combination with compatible genome design algorithms [14] this might allow integrated *in silico* and *in vivo* genome engineering for the first time.

## Conclusions

Combining *in silico* and *in vivo* research will soon be possible in genome engineering. With the release of increasingly refined whole-cell models, genome engineers will have an appropriate model, computational design algorithms, and scalable genetic editing technologies. Following the path of metabolic engineering, better design, and construction processes *in silico* and *in vivo* could be applied to a larger scale problem, replacing large quantities of lab work.

The next steps for genome engineering are: (i) the production and publication of new whole-cell models [154]; (ii) the implementation of computational standards to keep the field cohesive and prevent fragmentation [155]; (iii) the testing of *in silico* designs *in vivo* [14]; (iv) and the establishment of routine procedures for *in vivo* genome reductions for species that will soon have whole-cell models.

## Summary

- Metabolic engineering has an established process of *in silico* design and *in vivo* construction.
- *In silico* design informing *in vivo* construction is the future of genome engineering.
- Whole-cell models and algorithms for genome design will widen the field of genome engineering.
- Testing current *in silico* predictions *in vivo*, and uniting *in silico* and *in vivo* research in *E. coli*, are the next steps in genome engineering.

## Data Access Statement

The present study did not generate any new data.

## Abbreviations

CAGE: conjugative assembly genome engineering
COBRA: constraint-based reconstruction and analysis
FBA: flux balance analysis
FOCuS: flower pollination coupled clonal selection algorithm
GAMA: guess add mate algorithm
GACOFBA: genetic ant colony optimisation flux balance analysis
GDLS: genetic design through local search
GSMM/GEM: genome-scale metabolic model
KEGG: Kyoto encyclopedia of genes and genomes
MAGE: multiplex automated genome engineering
ME: macromolecular expression
MOMA: minimisation of metabolic adjustment
NOGD: non-orthologous gene displacement
NSAA: non-standard amino acid
ODE: ordinary differential equation
ROOM: regulatory on/off minimisation of metabolic flux
